# Active Tuning and Anisotropic Strong Coupling of Terahertz Polaritons in Van der Waals Heterostructures

**DOI:** 10.3390/mi13111955

**Published:** 2022-11-11

**Authors:** Shaopeng Li, Junhao Xu, Yajie Xie

**Affiliations:** 1 Department of Physics, Shaanxi University of Science and Technology, Xi’an 710021, China; 2 State Key Laboratory of Transient Optics and Photonics Technology, Xi’an Institute of Optics and Precision Mechanics, Chinese Academy of Sciences (CAS), Xi’an 710119, China

**Keywords:** polariton hybridization, active tuning, anisotropic propagation, dispersion engineering, strong coupling

## Abstract

Electromagnetic field confinement is significant in enhancing light-matter interactions as well as in reducing footprints of photonic devices especially in Terahertz (THz). Polaritons offer a promising platform for the manipulation of light at the deep sub-wavelength scale. However, traditional THz polariton materials lack active tuning and anisotropic propagation simultaneously. In this paper, we design a graphene/α-MoO_3_ heterostructure and simulate polariton hybridization between isotropic graphene plasmon polaritons and anisotropic α-MoO_3_ phonon polaritons. The physical fundamentals for polariton hybridizations depend on the evanescent fields coupling originating from the constituent materials as well as the phase match condition, which can be severely affected by the α-MoO_3_ thickness and actively tuned by the gate voltages. Hybrid polaritons propagate with in-plane anisotropy that exhibit momentum dispersion characterized by elliptical, hyperboloidal and even flattened iso-frequency contours (IFCs) in the THz range. Our results provide a tunable and flexible anisotropic polariton platform for THz sensing, imaging, and modulation.

## 1. Introduction

In conventional optics, the ultimate spatial resolution is fundamentally restained by the diffraction limit to roughly half of the incident wavelength [1,2], which prohibits fabricating deeply sub-wavelength scale devices. Especially in the terahertz (THz) spectral range, due to the longer wavelength, the footprints of the THz devices is always about hundreds of micrometers [3,4], which is not compatible with modern integrated optics. Polaritons are hybrid modes formed by light strongly coupled to collective excitations in matter, which offers a promising platform for the manipulation of light at the deeply sub-wavelength scale [5,6,7,8,9]. In THz range, gold is the commonly used polariton platform for confined THz techonlogies; however, it sustains low confinement factors, high transmission losses and lack of active tunability [10,11]. Graphene, a kind of two-dimensional material, presents THz plasmon polaritons (PPs) with high confinent factors and long lifetimes. Most importantly, the Fermi level can be conveniently adjusted by electrical gating [12,13]. The only existing shortcoming in graphene plasmon is the isotropic propagation. α-MoO_3_ is a kind of polar Van der Waals (vdWs) material; it is reported that the anisotropic phonon polaritons (PhPs) in mid-infrared and THz have been verified and can also be tuned by ion intercalation [14,15,16]. However, active tuning can not be executed. Therefore, a new kind of material or platform supporting THz polaritons with active tuning and anisotropic propagation is urgently needed. 

VdW heterostructures provide an attractive platform for integrating bulk materials into an atomic interaction level, giving rise to an extraordinary hybridization of different polariton modes [17]. In addition, the physical fundamentals for polariton hybridizations depend on the evanescent fields coupling originating from the collective charge oscillations in constituent materials. However, the prerequisites—phase match conditions—for polariton hybridizations must be satisfied: firstly, the constituent polaritons need to propagate in the same direction with mode volumes coinciding, and secondly, the individual polaritons must possess nearly the same momenta [18,19,20]. Consequently, hybrid polaritons propagate with in-plane anisotropy that exhibits momentum dispersion characterized by elliptical, hyperboloidal and even flattened iso-frequency contours (IFCs) in the THz range.

For that purpose, we assembled graphene/α-MoO_3_ heterostructures for hybridizing isotropic graphene PPs with anisotropic α-MoO_3_ PhPs, thus permitting substantial in-plane anisotropy to the plasmonic resonance of graphene. It is found that the polariton hybridization can be severely affected by the α-MoO_3_ thickness and actively tuned by the gate voltages. Moreover, the anisotropic momentum dispersion can be converted from elliptical IFCs to flattened bands. Finite difference time domain (FDTD) methods were used to simulate the mode field distributions and extract the polariton wavelengths. The anisotropic real-space near-field electromagnetic distributions were visualized by a phenomenological cavity model within a graphene/α-MoO_3_ nanostructure, which indicates that the electromagnetic field can be restricted into specific patterns depending on the illuminating frequency. Our results reveal a tunable and scalable anisotropic polariton platform for applications in sensing, imaging, and light modulation. 

## 2. Optical Conductivity and Dispersion Analysis

**Figure 1:**
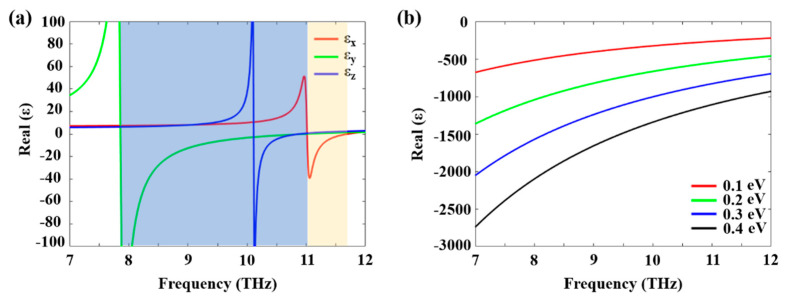
Dielectric permittivity of α-MoO_3_ and graphene in the THz spectral range. (**a**) The real parts of the permittivity tensor of α-MoO_3_ reveal two distinct in-plane RBs with negative permittivities along different crystal axes, shaded in blue and yellow RBs along [001] and [100] crystalline directions, respectively. (**b**) The real parts of graphene permittivity with different Fermi energies.

In THz, the optical response of α-MoO_3_ is dominated by the phonon absorption, and phonon polaritons (PhPs) in α-MoO_3_ can be excited within spectral intervals of the reststrahlen bands (RBs), in which the real part of the permittivity is negative. The permittivities of α-MoO_3_ in THz can be described by the following Lorentz equation [15]:

(1) $$\varepsilon_{j}=\varepsilon_{\infty ,j}\left(\frac{\omega_{LO,j}^{2}-\omega^{2}-i\omega \Gamma_{j}}{\omega_{TO,j}^{2}-\omega^{2}-i\omega \Gamma_{j}}\right)$$

where j=x,y,z denotes the three principal axes of α-MoO_3_ crystal, which corresponds to the crystalline directions [100], [001], and [010], respectively. $\omega_{TO,j}$ and $\omega_{LO,j}$ refer to the transverse-optical (TO) and longitudinal-optical (LO) phonon frequencies. Γ and $\varepsilon_{\infty ,j}$ are the damping constant and high-frequency dielectric constant, respectively. The detailed parameters used in calculations are given in reference [15]. As shown in Figure 1a, we observe two spectral bands (marked as shaded blue (low) and yellow (high) ranges) wherein at least one of the principal components of the permittivity is negative. In particular, the low- and high-frequency RB bands are located along the [001] and [100] crystal directions, respectively, indicating their potential to support PhPs with in-plane hyperbolic propagation.

Graphene’s surface conductivity is modeled using the Kubo formula [21,22]:

(2) $$\sigma_{g\;}=i\frac{e^{2}k_{B}T}{\pi \hbar^{2}\left(\omega +i\tau^{-1}\right)}\left[\frac{E_{F}}{k_{B}T}+2ln\left(\exp\left(-\frac{E_{F}}{k_{B}T}\right)+1\right)\right]+i\frac{e^{2}}{4\pi \hbar }ln\left[\frac{2\left|E_{F}\right|-\hbar \left(\omega +i\tau^{-1}\right)}{2\left|E_{F}\right|+\hbar \left(\omega +i\tau^{-1}\right)}\right]$$

Here, *e* is the electron charge, *E_F_* is the Fermi energy, ℏ is the reduced Planck constants, and *τ* is the momentum relaxation time. The relaxation time of charge carriers in graphene is set to 0.5 ps, which is typical for encapsulated graphene used in optical experiments at ambient conditions. The permittivity of graphene can be written as $\varepsilon =-\sigma /i\omega t_{g}\varepsilon_{0}$, where $t_{g}=0.34\;\mathrm{nm}$ is the thickness of graphene, and *ε*_0_ is the vacuum permittivity. The real parts of the permittivity tensors of graphene with different Fermi levels are negative in the frequency range from 7 THz to 12 THz (Figure 1b), which promises actively tunable PPs. 

## 3. Polariton Hybridization Analysis

**Figure 2:**
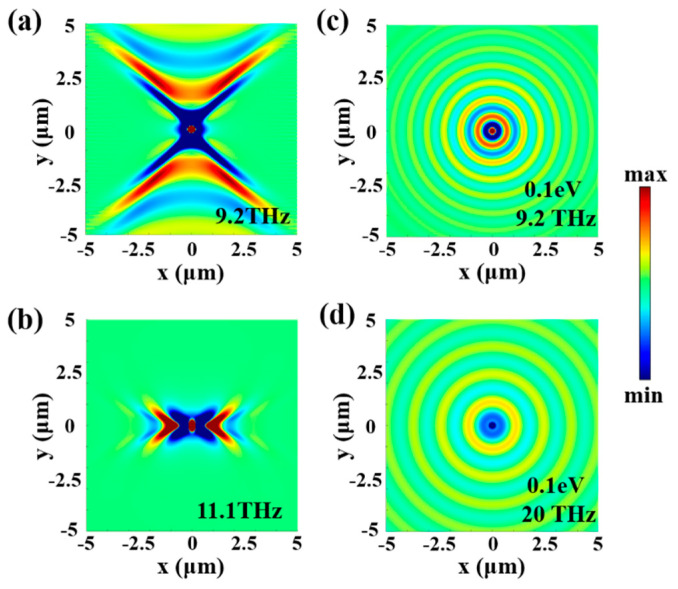
Predictions of THz polaritons in α-MoO_3_ and graphene with hyperbolic and circular propagation, respectively. (**a**,**b**) Images of the real part of the near-field amplitude distribution (E_z_) on top of a 20 nm α-MoO_3_ film; (**c**,**d**) real part of E_z_ on top of graphene under different excitation frequencies.

The FDTD method has been performed to investigate the polariton response of graphene and α-MoO_3_ in detail. A vertical dipole was placed above the top of the uppermost surface of 70 nm as a polariton launcher, the thickness of α-MoO_3_ film was set to 20 nm and the graphene layer was regarded as a surface conductivity sheet. The IFCs were then numerically obtained by fast Fourier transform (FFT) of the real part of the z component of the near-field amplitude E_z_. Due to the opposite in-plane permittivity signs of α-MoO_3_ along [001] and [100] crystal directions, polariton propagation along the [100] direction is forbidden at the low frequency band, which resulted in hyperbolic Ez distribution centered along the crystallographic directions of [001] (Figure 2a). In contrast, hyperbolic E_z_ distribution at the high frequency band is centered along the crystallographic directions of [100] (Figure 2b). The near-field images present highly anisotropic polaritons with frequency-dependent propagation. However, for PPs in graphene, polaritons are allowed to propagate along all possible in-plane directions, which results in circular E_z_ distribution (Figure 2c,d). Therefore, the hybridized polaritons will propagate along all possible in-plane directions with polaritons superpositioned between graphene PPs and α-MoO_3_ PhPs, which results in elliptical iso-frequency contours.

**Figure 3:**
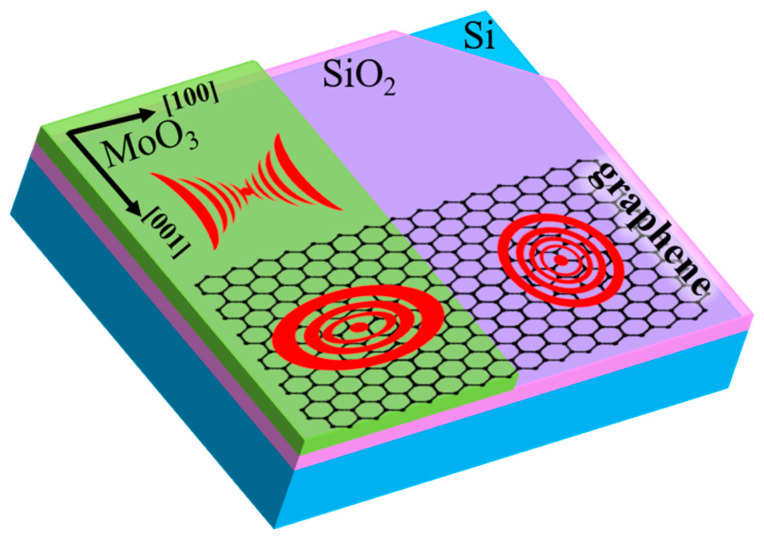
Schematic of the polariton hybridization in graphene/α-MoO_3_ heterostructure.

The permittivity results suggest the potential formation of hybrid plasmon-phonon polariton modes in two excitation bands. We thereby construct a platform for hybridizing isotropic graphene PPs with anisotropic α-MoO_3_ PhPs, thus resulting in anisotropic and tunable hybridized polaritons (Figure 3). However, the preconditions for polariton hybridization must be satisfied. In our analytical calculations and the following numerical simulations, the distance between the graphene layer and the α-MoO_3_ slab is zero; hence, the overlap between the graphene PPs and PhPs fields is maximal. Nevertheless, whether the PPs and PhPs couple or not is determined by whether there is a crossing point between the dispersion of PPs (without PhPs) and the dispersion of PhPs (without PPs). If there is a crossing point between the dispersions for a given configuration, there will be coupling. Therefore, the graphene Fermi level, α-MoO_3_ thickness, spectra range, and polariton momenta must be optimized. 

The dispersion of graphene plasmons are governed by a frequency (ω)—momentum (q) relation given by [23]:

(3) $$q_{\mathrm{SPP}}=\frac{i\omega \kappa }{2\pi \sigma_{2D}}$$

where $\kappa \left(\omega \right)=\frac{\varepsilon_{o}+\varepsilon_{sub}\left(\omega \right)}{2}$, $\varepsilon_{o}$ and $\varepsilon_{sub}$ are the vacuum and substrate permittivites, respectively, and $\sigma_{2D}$ represents the sheet conductance of graphene. In calculation of the plasmon dispersion in a graphene sheet embedded between a semi-infinite air space and a semi-infinite dielectric environment of α-MoO_3_, $\varepsilon_{sub}=\varepsilon_{\infty ,x}$ when calculating the dispersion along the [100] crystal axis and $\varepsilon_{sub}=\varepsilon_{\infty ,y}$ along the [001] crystal axis. The phonons of α-MoO_3_ are not considered in this calculation. The dispersion relation of electromagnetic modes in biaxial α-MoO_3_ slabs can be described by the following analytical approximations [24]:

(4) $$\;q=\frac{\rho }{k_{0}d}\left[arctan\left(\frac{\varepsilon_{1}\rho }{\varepsilon_{z}}\right)+arctan\left(\frac{\varepsilon_{3}\rho }{\varepsilon_{z}}\right)+\pi l\right],\;l=0,1,2\ldots \ldots$$

(5) $$\rho =i\sqrt{\frac{\varepsilon_{z}q^{2}}{\varepsilon_{x}q_{x}^{2}+\varepsilon_{y}q_{y}^{2}}}=i\sqrt{\frac{\varepsilon_{z}}{\varepsilon_{x}cos^{2}\varphi +\varepsilon_{y}sin^{2}\varphi },}$$

where φ is the angle between the *a* axis and the in-plane wave vector *q* ($q^{2}=q_{x}^{2}+q_{y}^{2}$), $\varepsilon_{1}$ and $\varepsilon_{3}$ are the dielectric constants of the superstrate and substrate of the α-MoO_3_ slab, *k_0_* and *d* are the free-space wave vector and α-MoO_3_ thickness, respectively, and $\varepsilon_{x}$, $\varepsilon_{y}$ and $\varepsilon_{z}$ are the permittivities of α-MoO_3_ along *a*, *b* and *c* axes, respectively. 

**Figure 4:**
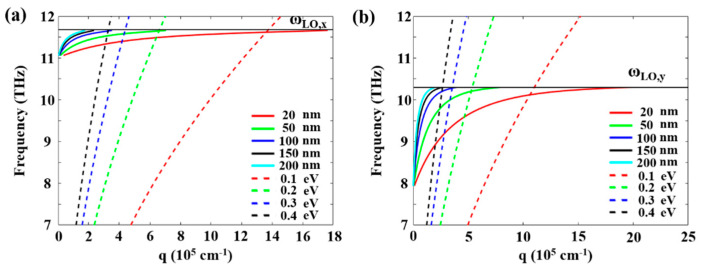
Analytical dispersion of polariton in graphene and α-MoO_3_. Dispersion of PhPs in bare slabs of α-MoO_3_ with different thicknesses surrounded by vacuum (solid lines), as well as the dispersion of PPs in a graphene sheet surrounded by a semi-infinite air and a semi-infinite dielectric environment of α-MoO_3_ for different Fermi energies (dashed lines). (**a**) Dispersion along [100] and (**b**) [001] crystal directions.

Figure 4a,b shows the dispersion for graphene PPs with the suppressed α-MoO_3_ PhPs for different Fermi energies (dashed lines), as well as the dispersions of PhPs in the α-MoO_3_ slabs for different crystal thicknesses (solid lines). We can observe that the higher the Fermi energy, the wider the range of thicknesses of the biaxial slab for which there exists coupling between PPs and PhPs. If the slab is too thick, then a higher Fermi energy is required to match the PPs wavevector and achieve the coupling regime. On the contrary, when the slab is thinner, a lower Fermi energy is required. Significantly, for thinner slabs (20 nm), a wider range of graphene PPs will couple with α-MoO_3_ PhPs, which permits larger polariton coupling and active tunable range. Therefore, in the following calculations, the thickness of α-MoO_3_ is fixed at 20 nm. 

**Figure 5:**
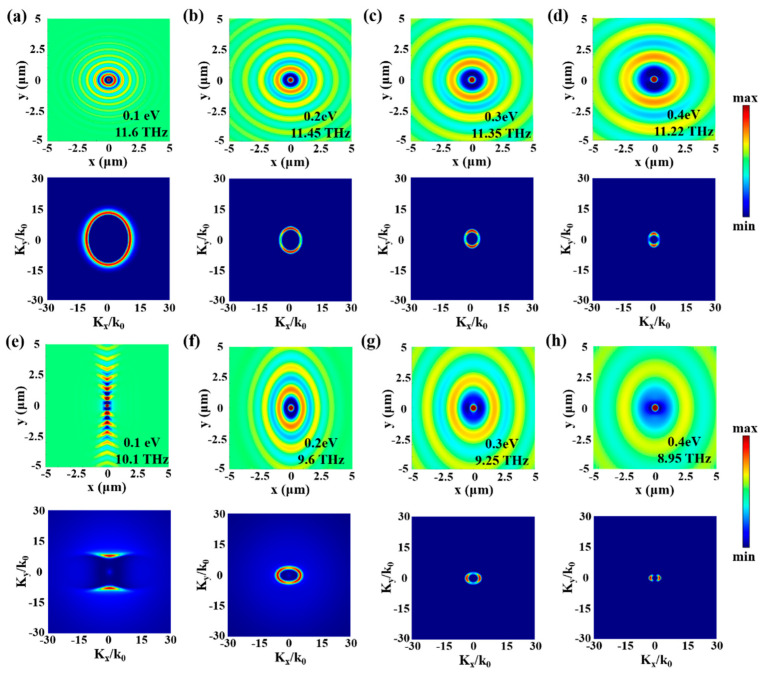
Hybrid polariton evolution in graphene/α-MoO_3_ heterostructure as a function of frequency and Fermi energy. (**a**–**d**) Simulated electric field spatial distribution (**top panels**) and corresponding colorplots IFCs (**bottom panels**) of hybrid polaritons at high-frequency bands for Fermi energies of 0.1, 0.2, 0.3, and 0.4 eV, respectively. (**e**–**h**) Electric field spatial distribution (**top panels**) and the corresponding colorplots IFCs at the low-frequency band.

To study in more detail the polariton-coupling features as a function of Fermi energy in graphene/α-MoO_3_ heterostructures, we calculate the E_z_ distribution at the crossing-point frequencies in Figure 4. When the graphene Fermi energy is 0.1 eV, the polariton coupling should be enhanced at 11.6 THz; the near-field E_z_ distribution and the corresponding FFT are shown in Figure 5a. It is found that the near-field distribution features elliptic wavefronts with long axes centered along the crystallographic directions [100], which is significantly different from that typically observed in bare graphene or α-MoO_3_ (Figure 2). By increasing the Fermi energy to 0.2 eV, the polariton coupling is enhanced at a lower frequency of 11.45 THz, which also manifests elliptic electric field wavefronts. However, the hybrid polariton wavelength significantly increased, with the colorplot of FFT centered in a narrower wavevector range. With the Fermi energy further increased to 0.3 eV, coupling frequency is shifted to 11.35 THz with polariton wavelength increased further. The evolutionary process is also the same for 0.4 eV, with the hybrid polaritons enhanced at 11.22 THz with increased wavelength as well as smaller wavevector. 

For hybrid polaritons at the low-frequency band, more interesting phenomena present. When the Fermi energy is 0.1 eV, the polaritons are strongly hybridized at 10.1 THz, and the hybrid polaritons propagate in a specific direction (canalization regime). Note that the IFC exhibits a strong flattening along the [001] direction, explaining the canalization of hybrid polaritons along this direction (Figure 5e). When the Fermi energy increases to 0.2 eV, the near-field distribution features elliptic wavefronts with long axes centered along the crystallographic directions of [001], which is opposite to that for the high-frequency band. With the Fermi energy further increased to 0.3 eV and 0.4 eV, the hybrid polaritons also manifest elliptic wavefronts. However, the polariton wavelength increases with Fermi energies, and the corresponding colorplot of FFT centers in a narrower wavevector range. In addition, the hybrid polaritons manifest deeply sub-wavelength confinements of about 60 (λ_0_/λ_hybridized_) with respect to the incident THz wave (λ_0_ = 25.8 μm), which provides a nanoscale “hot spot” for enhancing light-matter interactions.

## 4. Manipulation of the Field Localization

The near-field simulations review found that the hybrid polaritons in graphene/α-MoO_3_ heterostructure can be strongly enhanced, and more importantly, the hybrid polaritons can be actively tuned from elliptic to flattened wavefronts by electrical gating. Moreover, the precise control of light localization is very important in nanophotonics, and the electromagnetic field concentration can be further enhanced by fabricating nanostructures that act as Fabry-Perot resonators. For this purpose, we precisely shaped graphene/α-MoO_3_ heterostructures into square nanostructures for tailoring electromagnetic field localizations. We adopted a phenomenological cavity model to simulate the near-field amplitude and phase patterns within graphene/α-MoO_3_ nanostructures [25,26]. A point dipole source launches circular surface waves and excites hybrid polariton waves in graphene/α-MoO_3_ nanostructures, the excited polariton wave propagates outward and is reflected by the nanostructure boundaries. The dipole-launched polariton waves interfere with the reflected waves perpendicular to the boundaries. The total field detected can be described as:

(6) $$E=E_{0}+\underset{j}{\sum }E_{p,j}$$

where *E*_0_ and *E_p,j_* are the dipole-launched polariton wave and that reflected from four boundaries, respectively. Reflected waves are given by $E_{p,j}=R_{j}\times E_{0}\times exp\left(-\left(\frac{4\pi }{\lambda_{p}}\right)r_{j}\left(\gamma_{p}+i\right)\right)$, where $R_{j}=R_{0}\exp\left(i\Delta \varphi \right)$ denotes the complex reflection coefficient, and *R*_0_ and Δ*φ* are the reflectivity and phase shift of the polariton wave, respectively. *γ_p_*, *r_j_* and *λ_p_* represent the polariton damping rate, distance between the nanostructure edge and dipole tip, and the polariton wavelength, respectively. In simulations the amplitude of the dipole-launched polariton wave *E*_0_ was set to 1 and the damping rate *γ_p_* to 0.4 eV, whereas the polariton wavelength *λ_p_* is set based on Figure 5. Reflectivity was assumed to be 1, and 1.5*π* was used for the phase shift of the polariton wave [26]. 

**Figure 6:**
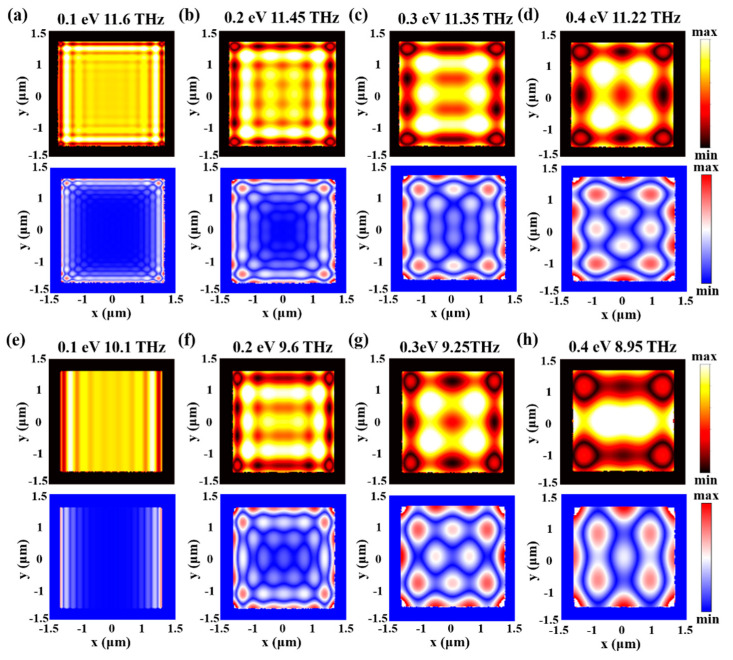
Near-field interference patterns of hybrid polaritons in graphene/α-MoO_3_ heterostructure micro-flakes. (**a**–**d**) The near-field amplitude interference patterns (**top panel**) and phase images (**bottom panel**) on top of the graphene/α-MoO_3_ micro-flakes at the high-frequency band. (**e**–**h**) The near-field amplitude and phase images on top of the graphene/α-MoO_3_ micro-flakes at the low frequency band.

Figure 6 shows the simulated near-field patterns of the hybrid polaritons in graphene/α-MoO_3_ micro-flakes at the cross points in Figure 4. Due to the multi-beam interferences caused by additional reflections from the square boundaries, near-field patterns of the hybrid polaritons give rise to a variety of bright and dark features as a function of exciting frequencies and Fermi energies. It is obvious that the bright (dark) spots resulting from constructive (destructive) interferences exhibit a D4-group symmetry. Both the amplitude and phase images manifest standing wave patterns, whereas the adjacent bright and dark fringes close to the boundaries differ by ~π/2. In addition, due to the damping of the reflected plasmon waves, more fringes with much weaker intensities can be observed in the interior part with the adjacent fringe separation of 0.5 *λ_p_*. 

In addition, due to the elliptical-type dispersion, hybrid polaritons propagate along *x* and *y* axes at different wavelengths. Except for the flattened-band dispersion at 10.1 THz, polariton propagation along *y* axis is forbidden, which results in interference fringes only appearing in the horizonal direction. As the frequency decreases, the number of bright spots decreases and the field localizations are strongest at the regions close to the corner of the nanostructures. The realization of electromagnetic field localization in two dimensions using graphene/α-MoO_3_ nanostructures can offer several advantages in terms of efficient focusing and manipulating of light at the nanoscale, which can further be integrated with photonic or optoelectronic devices to enhance light-matter interactions. 

## 5. Conclusions

In conclusion, we designed and investigated polariton hybridizations in graphene/α-MoO_3_ heterostructures. It was found that the polariton hybridization between isotropic graphene PPs and anisotropic α-MoO_3_ PhPs strongly depends on the α-MoO_3_ thickness and can be actively tuned by the gate voltages. In addition, the hybrid polaritons propagate with in-plane anisotropy that exhibits momentum dispersion characterized by elliptical, hyperboloidal and even flattened IFCs in the THz range. The near-field results suggest that the localized electromagnetic fields can be enhanced in nanostructures, which provide a flexible anisotropic polariton platform and can further be integrated with photonic crystals or quantum well structures to ulteriorly enhance THz-matter interactions. Our findings demonstrate that active tuning of THz polaritons with certain propagations can be used to steer THz light along specific directions at the nanoscale. More significantly, these results will provide guidelines for THz device fabrications and experiments with dynamically controllable properties.

## Data Availability

The data presented in this study are available on request from the corresponding author.
